# Exploring pregnancy termination experiences and needs among Malaysian women: A qualitative study

**DOI:** 10.1186/1471-2458-12-743

**Published:** 2012-09-05

**Authors:** Wen Ting Tong, Wah Yun Low, Yut Lin Wong, Sim Poey Choong, Ravindran Jegasothy

**Affiliations:** 1 Medical Education and Research Development Unit, Faculty of Medicine, University of Malaya, 50603, Kuala Lumpur, Malaysia; 2 Dean’s Office, Faculty of Medicine, University of Malaya, 50603, Kuala Lumpur, Malaysia; 3 Department of Social and Preventive Medicine, Faculty of Medicine, University of Malaya, 50603, Kuala Lumpur, Malaysia; 4 Klinik Rakyat Family Planning Services SdnBhd, 556-W, Mk 13, BatuUban, Century Garden, 11700, Gelugor, Penang, Malaysia; 5 Department of Obstetrics & Gynaecology, Hospital Kuala Lumpur, Jalan Pahang, 50586, Kuala Lumpur, Malaysia

**Keywords:** Abortion, Experiences, Needs, Unwanted pregnancy, Women's health, Sexual health, Family planning, Reproductive health, Interview

## Abstract

**Background:**

Malaysia has relatively liberal abortion laws in that they permit abortions for both physical and mental health cases. However, abortion remains a taboo subject. The stagnating contraceptive prevalence rate combined with the plunging fertility rate suggests that abortion might be occurring clandestinely. This qualitative study aimed to explore the experiences of women and their needs with regard to abortion.

**Methods:**

Women from diverse backgrounds were purposively selected from an urban family planning clinic in Penang, Malaysia based on inclusion criteria of being aged 21 and above and having experienced an induced abortion. A semi-structured interview guide consisting of open ended questions eliciting women’s experiences and needs with regard to abortion were utilized to facilitate the interviews. Audio recordings were transcribed verbatim and analyzed thematically.

**Results:**

Thirty-one women, with ages ranging from 21–43 years (mean 30.16 ±6.41), who had induced surgical/medical abortions were recruited from an urban family planning clinic. Ten women reported only to have had one previous abortion while the remaining had multiple abortions ranging from 2–8 times. The findings revealed that although women had abortions, nevertheless they faced problems in seeking for abortion information and services. They also had fears about the consequences and side effects of abortion and wish to receive more information on abortion. Women with post-abortion feelings ranged from no feelings to not wanting to think about the abortion, relief, feeling of sadness and loss. Abortion decisions were primarily theirs but would seek partner/husband’s agreement. In terms of the women’s needs for abortion, or if they wished for more information on abortion, pre and post abortion counseling and post-abortion follow up.

**Conclusions:**

The existing abortion laws in Malaysia should enable the government to provide abortion services within the law. Unfortunately, the study findings show that this is generally not so, most probably due to social stigma. There is an urgent need for the government to review its responsibility in providing accessible abortion services within the scope of the law and to look into the regulatory requirements for such services in Malaysia. This study also highlighted the need for educational efforts to make women aware of their reproductive rights and also to increase their reproductive knowledge pertaining to abortion. Besides the government, public education on abortion may also be improved by efforts from abortion providers, advocacy groups and related NGOs.

## Background

In 2007, the Millennium Development Goal target 5b, ‘universal access to reproductive health’ was integrated, aiming to empower girls and women in exercising their sexual and reproductive rights. However, women’s control of their reproductive health and life, in deciding when to have children, the number and spacing of their children are still restricted. These restrictions are linked to the laws governing abortion as well as structural, religious and cultural conservatism, religious fundamentalism and social stigmatization that serve as barriers for women to access abortion services when they require it. Therefore they had to resort to unsafe methods. When women are denied the rights to control their fertility, it will lead to an unsafe abortion.

In 2008, it was estimated there were 21.6 million unsafe abortions worldwide. Annually, 68,000 of women die due to complications following unsafe abortions [1]. The world sees 13% of women dying because of inaccessibility to safe abortion services [2]. In the Asian region, 10.8 million unsafe abortions were estimated with the unsafe abortion rate at 11 per 1000 women aged 15–44 years [3].

Different countries have different permissible grounds in which abortion can be performed legally [4]. Developed regions of the world (Europe, Northern America, Australia/New Zealand and Eastern Asia) have less restrictive laws and policies with regard to abortion as more than half (67%) of the developed countries give full abortion decision/rights entirely to women. In contrast, less developed regions (Africa, Western Asia, South-Central Asia, Central America, South-Eastern Asia, South America and the Carribbean region) are stricter with only 15% of developing countries provide abortion under ‘upon request’ [4]. Most unsafe abortions (97%) occur in developing countries [5].

### Abortion in Malaysia

In Malaysia, the total fertility rate plunged from 3.0 in 2000 to 2.3 in 2008 even though the contraceptive prevalence rate has stagnated for the past 20 years [6]. This may suggest that abortions occurs widely in the country but yet no official statistics can be found for abortion rates in Malaysia. Nevertheless, Tey (2010) using Bongaart’s model estimated that the abortion rate to be 16% [6] in Malaysia.

Malaysia has a less restrictive law, whereby the law permits abortions to be carried out if the pregnancy poses a risk to the life of the pregnant woman or is injurious to the physical and/or the mental health of the pregnant woman (Section 312, Penal Code), however, access to abortion services remains limited.

The public health sector provides reproductive health services to all but contraceptive services are only provided to married women. While private abortion services are widely available, they are generally discreet, often unregulated and charge exorbitant prices, hence making it inaccessible to those of lower economic status. Due to the lack of a policy guideline on abortion services by the Ministry of Health, the practice of abortion depends on the views of the individual practitioners at the public health institutions. As a consequence, sometimes women are required to follow non-uniform procedures for example, psychiatric tests, the need for a second medical opinion and even obtaining the husband’s consent when the law does not impose such a burden. The fact that doctors are obliged to follow these procedures coupled with personal judgments further compound the women’s access to abortion services. This would cause unnecessarily delays in women’s access for abortion [7].

Surgical methods form the mainstay of abortion services in the public sector. Dilatation and curettage was the main method used often on an inpatient basis. Ambulatory care methods of abortion provision came into use with the establishment of early pregnancy assessment units (EPAU) in some major hospitals. The use of the Karman cannula and prostaglandins came into use in selected hospitals which were in a minority. The traditional D&C still forms the main method used in the vast majority of cases.

Abortion services were mainly provided for the range of missed and incomplete abortions that were presented to the public hospitals. Terminations of pregnancies were mainly performed for medical reasons and never on request. The use of misoprostol and mifepristone have not been sanctioned for abortions in the Ministry of Health.

Abortion is considered taboo in Malaysia and studies on issues surrounding abortion are rare. Suggestions that abortions might be occurring clandestinely has prompted this study to explore the experiences and needs of women who had experienced induced abortions in Malaysia. This qualitative study aimed to explore the experiences of women and needs with regard to abortion. It is hoped that the findings of this paper will help to understand the current situation with regard to abortion in this country and benefit and promote the reproductive well being and ultimately the quality of life of women in this country.

## Methods

This study adopted a qualitative approach (one-to-one interview) in order to gain an in-depth understanding by uncovering values, the meaning behind the experiences of women who have had an induced abortion and as well as their needs with regard to abortion. Women from diverse backgrounds were purposively selected from an urban family planning clinic in Penang, Malaysia.

The clinic operates as ambulatory care centre providing outpatient contraceptive and gynaecological services. It provides a full range of modern contraceptive methods including COCs, DepoProvera injections, IUCD insertions and vasectomy. It also offers first trimester terminations both surgical (manual vacuum aspiration under local anaesthesia) as well as medical abortion (methotrexate and misoprostol). While it has been in operation legally since 1989, the Ministry of Health, acting on the regulations of the Malaysian Private Healthcare Facilities and Services (PHFS) Act 1998, has recently questioned the safety of providing MVA terminations in a primary care environment; this matter has still not been resolved. As abortions services are still stigmatized within the medical community, despite its legal status, this clinic was the only one willing to participate in research on the subject of voluntary abortions.

Data collection was conducted during the period May to June, 2011. The patients’ medical records were searched for women who fitted the inclusion criteria of the study; those who experienced induced abortion (surgical and/or medical abortion). Such women needed to be aged 21 and above. Walk-in patients who fulfilled the inclusion criteria were also approached to participate.

A semi structured interview guide consisting of open ended questions was constructed based on the literature review and discussion among researchers. This was utilized to gather verbatim data from the respondents. Issues probed included women’s experiences with abortion services, their feelings about them in terms of information, education on different abortion methods, medical care, safety of procedure, post-abortion medical care, supportive care, such as pre- and post-abortion counseling, cost of procedure and follow-up care. Issues on abortion decision, support received, factors assisted or hindered in coping with abortion, motivators and barriers in seeking for abortion services were also explored. Opinions of the abortion service providers were also asked. In terms of needs, the women were asked on what information women want on abortion, and what can be done to help women to cope with abortion.

This study obtained the ethics approval from the University of Malaya Medical Centre Medical Ethics Committee, Kuala Lumpur, Malaysia.

All interviews were carried out in the clinic where confidentiality and privacy was ensured. Prior to the interview, the women were told about the objectives of the study. It was stressed that participation was voluntary and that confidentiality would be maintained. A study information sheet was provided which detailed the potential risks, benefits and the rights of the patients. Verbal and written informed consent was obtained from all respondents. A short questionnaire was administered to obtain socio-demographic information of the respondents. The interviews were audio recorded (with permission from the respondents).

The audio recordings were transcribed verbatim. Malay and Mandarin transcripts were translated to English. Malay transcripts were translated by researchers themselves while Mandarin transcripts were translated to English by hired persons who are bilingual in Mandarin and English. Subsequently, the transcripts were checked by researcher (TWT). Translated quotes in the Results section are italized.

A qualitative data software manager, NVIVO 9 was used. Thematic analysis which is derived from grounded theory was utilized. Initially, line by line of transcripts was read and coding was performed by assigning a short specific description which characterized a particular statement response by participants. This was done for the first 3 transcripts which led to the development of a coding framework. The coding framework was subsequently used for coding of the remaining 28 transcripts. Any disagreements during the analysis process were discussed and themes were reached after consensus was achieved among the researchers. This counteracted possible researcher bias. The findings emerged from the analysis were illustrated by quotes and this is to ensure that the themes are representative of the data to avoid misinterpretation.

## Results

Forty-five women who passed the criteria for study inclusion were approached for participation and 31 agreed to be interviewed. Among the 14 women who rejected participation, reasons were reluctance to discuss the study topic, unavailability for the planned interview as well as time constraints, disapproval from husband/partner to participate and concerns with confidentiality.

The socio-demographic profile of the 31 women who participated is shown in Table 1. These women had their last induced abortion between July 2010 and May 2011 and all of their last abortions were medical in nature. Ten to Nine women reported that it was their first time having an induced abortion. The remaining had experienced more than one induced abortion with some having gone through both surgical and medical abortions.

**Table 1:** Background characteristics of the women

**Characteristics**	**Number (n = 31)**	**Percent (%)**	**Mean ± SD (Range)**
**Age (years)**			30.16 ± 6.41 (21–43)
**Ethnicity**			
Malay	16	51.6	
Chinese	10	32.3	
Indian	5	16.1	
**Religion**			
Islam	16	51.6	
Buddhist/Taoist	10	32.3	
Hindu	5	16.1	
**Education level**			
Primary	2	6.5	
Secondary	23	74.2	
Tertiary	6	19.4	
**Marital status**			
Single	5	16.1	
Married	25	80.6	
Divorced/Separated	1	3.2	
**Monthly Personal Income**^**1**^			
≤ RM 1000	7	29.2	
RM1001-RM2000	15	62.5	
RM2001-RM3000	0	0	
RM3001-RM4000	1	4.2	
RM4001-RM5000	1	4.2	
**Monthly Household Income**			
≤ RM 1000	2	6.5	
RM1001-RM2000	11	35.5	
RM2001-RM3000	1	3.2	
RM3001-RM4000	11	35.5	
RM4001-RM5000	5	16.1	
RM5001-RM6000	1	3.2	
**Occupational group**^**2**^			
Technicians & Associates professional	2	6.5	
Clerical support worker	7	22.6	
Service and sales worker	7	22.6	
Plant & machine operators & assembler	5	16.1	
Self-employed/freelance	3	9.7	
Housewife	7	22.6	

1 N = 24. Total is less as 7 women are housewives with no personal income.

2 Based on Malaysia Standard Classification of Occupations 2008, Ministry of Human Resource, Malaysia.

3 USD ($) 1 = Ringgit Malaysia (RM) 3.1.

Themes that were generated from the data analysis comprised of: personal abortion experiences, experiences towards accessing abortion services and information, fear about abortion and its side effects, feelings and emotions post-abortion, abortion decision making and support, and needs of women to cope with abortion.

### Personal abortion experiences

Most of the women claimed that medical abortion was similar to having normal menstruation with stomach cramps but few pointed out that they experienced intense pain and longer bleeding duration than the usual menses.

“Not very pain.(The bleeding) at first bit darker in colour, and after that it was normal – like the usual period, red in colour. When I was bathing, there were some solid little pieces, but there wasn’t a lot, some were like 5 cents [in size], some were like 20cents [in size]” **(29yrs_Chinese_secondary education level_single_admin clerk)**

“Very painful.I cannot bear even after few minutes. Then you can feel it [foetus] you know when suddenly it falls down. You can feel that it is no longer alive you know”** (23yrs_Malay_tertiary education _married_clerk)**

Most of them preferred medical abortion compared to surgical methods (D&C/MVA) as it took up less time, was cheaper, was more convenient and could easily be done at home, with little or no pain like normal menstruation. Women with impressions that surgical abortion would be painful, weaken the body and impose the need to stay in the clinic for post-abortion monitoring influenced some women to choose medical abortion.

“Can say that [that she opted medical abortion] because I scared to have surgical abortion and it is more expensive, so take medicine is much cheaper” (**26yrs_Chinese_secondary education _married_salesperson)**

In contrast, women who preferred surgical abortion (MVA) reasoned that they did not have to see or go through bleeding and pain. In addition, complete abortion can be achieved at the end unlike medical abortion which was perceived to be time consuming and completeness could not be assured.

“Surgical abortion is OK because after he washed all the blood inside, only some left. So, we won’t feel the stomach pain. Is easier because we will not be scared as we don’t see the clotted blood comes out because the doctor cleaned everything inside. Is just some spotting. That is usual after the procedure”**(26yrs_Malay_tertiary education_married_clerk)**

“Because I eat the pills, the menstruation is very heavy. ,I came to check that time they say cannot [incomplete abortion], need to do abortion [MVA] again., Did it twice, very inconvenient .If you do [surgical] abortion at the first time, you are ok when you go back”** [43yrs_Chinese_secondary education_married_housewife]**

### Experiences towards accessing abortion services and information

The main barriers to abortion were the lack of abortion services and information. Most of them could only obtain abortion information and service by asking friends or colleagues. They further noted that the information was often superficial and inadequate. Information obtained through the media was rare. Difficulties in obtaining information caused anxiety in women seeking abortion services and also to search for detailed information to make an informed decision. They were not sure which doctor would offer the service as not all doctors provided this service. One woman narrated that she had gone to few clinics before finally finding a clinic that provided such a service. Women in this study had the perception that government hospitals do not provide abortion services as it was illegal and thus did not make an effort to ask for this service in government hospitals. Some revealed that since they could not find the service, they had to resort to using traditional abortifacients. They did claim that they were not effective. Medical abortion was also noted to be uncommon.

“For me is very difficult. If you got pregnant, if you want to do abortion, you cannot go to government clinic or hospital. You can only come here [private clinic]”** (38yrs_Indian_secondary education_married_housewife)**

“No I didn’t go anywhere. Because if go to government [hospital], need to give birth to the baby. Cannot have abortion in government hospital, because they are very strict, conservative, very traditional mind, say abortion is illegal” **(29yrs_Chinese_secondary education_single_admin clerk)**

Since abortion was a controversial issue and considered a taboo, women who sought abortion information as well as the services had difficulties in requesting for them directly. With regard to abortion services, one woman claimed that she was asked for a pregnancy scan first and then found out about abortion. In addition, the women also felt that it was embarrassing to ask information about abortion service as they ‘knew’ it is a sin and feared other people would talk, behind their back. A single woman claimed that she did not go back to the previous clinic where she had her first abortion as she felt embarrassed to let people know that she was going for her second abortion.

“Is embarrassing to tell them because we know all these things are sinful. We tell them we want to abort. Later, there will be people who will talk behind our back”** (22yrs_Malay_secondary education_married_housewife)**

“(laughing) I already had [abortion] before, I don’t want to go back. They will say this girl come again. Because they got my profile. So, I don’t want [to go back to the same clinic again]” **(25yrs_Chinese_secondary education_single_hairstylist)**

Cost did not seem to affect women’s access to abortion. Since all the women interviewed had obtained abortions in the clinic where they were recruited, the majority felt that the abortion fee charged was reasonable and commensurate with the services they received. However, there were some who found the fee to be high especially surgical abortion (RM400-RM700) which was more expensive compared to medical abortion (RM150-RM260). The cost influenced some women to opt for medical abortion as it was cheaper. One woman felt that no matter what the cost was for abortion, women were compelled to pay due to desperation to terminate the unwanted pregnancy.

“Yeah, money is a problem because my husband is the only one working. I’m housewife with three children and also I have to support my father-in-law. I take pills because first time I did washing [surgical abortion] it cost almost RM500”**(38yrs_Indian_secondary education_married_housewife)**

“But if someone wants to abort. No matter how expensive it is, they are willing [to pay]”**(35yrs_Malay_secondary education_married_nasilemak seller)**

Pertaining to abortion services in the clinic, the women were satisfied with the service given particularly towards the detailed and clear information received on the types of abortion offered, systematic information on the processes for medical abortion and the emphasis on post-abortion follow-up care. The follow-up care involved ultrasound scanning to ensure completeness following medical abortion. The patients were appreciative that they were not charged but instead were refunded for the remaining medications that they did not need, if the abortion was complete.

With regard to health providers in the clinic, many were satisfied and felt they were able to discuss with the doctors on the options and had time to decide on abortion and contraception. Unmarried women were appreciative of the non-judgmental treatment received from the clinic. Several women had negative experiences in other clinics; one unmarried women claimed that she faced resistance in trying to enquire for abortion service while another women compared the kind and positive attitudes of health providers in the clinic with those from other clinics whom they noted were ‘clinical’. With regard to government hospitals/clinics when seeking abortion services, the women felt unhappy as the doctors were judge mental with their personal beliefs that abortion is wrong and sinful. They were told off, that abortion was against religion and were asked to carry their pregnancy to term and later put up the baby for adoption.

“I am already pregnant so the doctor wants to set up antenatal care appointment. I said ‘Wait doctor’. I went with my husband. ‘My children are still young, wait’. Doctor scolded ‘Cannot abort. Is wrong”** (28yrs_Indian_secondary education_married_part time driver)**

“’Why you want to throw [abort]? You know that is a sin right’, the doctor said”** (22yrs_Malay_secondary education_married_housewife)**

The women were also pleased with the pre- and post- abortion counseling that they received as part of its comprehensive abortion services. They felt the pre-abortion counseling was not prejudiced and judgmental but served to provide information and clarified fears/doubts and misinformation. However, some felt that there was a lack of details about abortion. Some women appreciated that the pre- and post-abortion counseling involved their partners.

### Fears about abortion and its side effects

The major fears experienced by women were primarily incompleteness of the procedure and future infertility. Other fears were safety issues and uncontrolled bleeding. There were doubts about the effectiveness of medical abortion and worries about the potential harmful ingredients in the medication. With regard to surgical methods/MVA, there were fears that the procedure would weaken the body.

“After the procedure, I have thought about whether the baby is completely washed out. When I was checking, I felt a little bit nervous and I was afraid that the baby couldn’t be completely washed out”** (29yrs_Chinese_secondary education_single_admin clerk)**

“I have heard people said if have abortion, then is hard to get pregnant again (in the future)”** (22yrs_Malay_secondary education_married_housewife)**

“Doctor said by taking this medicine, 99% can come out. At first, I don’t believe. I know that my body is strong. It won’t come out. Can it be aborted by taking medicine? I am surprised”** (28yrs_Indian_secondary education_married_part time driver)**

The women reported that they had not experienced any side effects but only normal post-abortion effects. Nevertheless, some were still concerned about infections and future consequences such as possible infertility, damaged uterus or reproductive cancer resulting from surgical methods. There was also a fear of giving birth to a handicapped child or a child will be born with some abnormality in the future.

“Like maybe virus or bacteria .Sometimes, the instruments that they use may cause infection in our womb. Scared if it affects my future pregnancy”** (31yrs_Malay_tertiary education_married_clerk)**

“After abortion, doctor won’t encourage us to get pregnant in 2 years. They are afraid if the child will be different [abnormal]”** (22yrs_Malay_secondaryeducation_married_housewife)**

“[after surgical abortion] As for the future. is quite worrying. scared if the uterus is damaged, or if there is cancer or what. But if through medicine [medical abortion], for me it is safe because it doesn’t involve anything”** (22yrs_Malay_secondaryeducation_married_housewife)**

Only one woman showed confidence with modern abortion methods and believed that it should be safe.

“Because now, I think the technology has improved already. So, I am not scared of any side effects”** (25yrs_Chinese_secondaryeducation_single_hairstylist)**

### Post-abortion feelings and emotions

Abortions brought a myriad feelings to women who had experienced it, from seemingly “no feelings”, not wanting to think about the abortion, relief, feeling of sadness and loss.

“I think I don’t have any feelings. No, I didn’t feel (the) loss or anything. Not at all. I didn’t think about this. I didn’t think that thoroughly”** (38yrs_Chinese_primary education_married_factory QA)**

“For me, abortion.I feel satisfied, a relief”** (35yrs_Malay_secondary education_married_nasi lemak seller)**

“Oh.you see it comes out [foetus], feels very sad, really sad”** (23 yrs_Malay_tertiary education_married_clerk)**

The women also reported feelings of post-abortion regret and this feeling came from the guilt of aborting the baby. This feeling was related to the cultural and spiritual ties of the women as they felt they had sinned against God. There were also few women who felt guilty towards the unborn baby. Some of them coped with these feelings through prayers, asking forgiveness from God and the unborn fetus.

“One month after the wash [abortion], we already sinned. After that, I went to ask for forgiveness. Me and my husband and children bring. [to temple] let him [the unborn foetus] know, asked for forgiveness. The child [unborn foetus] also has no life right. I asked for forgiveness from him [the aborted foetus]. Whether he knows or not I don’t know. But I aborted, the baby I abort right. Please forgive”** (28yrs_Indian_secondaryeducation_married_part time driver)**

“Feels guilty. Is between us and the One above. We know we already made a huge sin but we hope He (God) will understand our situation. Not like we simply just want to do this [abortion] right? People said it’s really such a waste. Usually we prayed. Increase our prayers, asked for forgiveness from the Above. Forgive our sin. We didn’t do it on purpose”** (22yrs_Malay_secondaryeducation_married_housewife)**

“I am a Buddhist. Everything, every single thing also has a soul although it hasn’t taken shape yet. It is also a life because it’s a life so I blamed myself. Before the abortion, even a dot is also a life. I told the baby it’s not I don’t want you but life forces people to do things. I have no choice but to abort you”**[29yrs_Chinese_secondaryeducation_single_admin clerk]**

### Abortion decision making and support

Majority of the women declared that the decision to abort was theirs but some had to persuade their partners about the decision. Although some initially reported that it was a joint decision, they later revealed that it was them (the women) who first decided and got their husbands to agree later. One woman stated that the decision to abort should be between husband and wife and should not involve other people (e.g. family member) as that would only add confusion to the decision making.

“Don’t know. Some they gave positive [opinion], some negative. Those I don’t want to listen, I discussed with my husband. Husband said ok, direct [straight for abortion]”** (28yrs_Indian_secondaryeducation_married_part time driver)**

As for those who were not married, the decision to abort was not because of her unmarried status but in not wanting to marry her boyfriend whom she thought was not the one for her. Another single woman claimed that she was forced to abort as her boyfriend claimed that he was not ready for marriage nor was he financially capable to support a family.

“First time I aborted because so young and then that guy, I don’t think he is my man. So the second time [when I was pregnant] my friend told me, “Now with your age, you also can get married. Why don’t want you want to marry”. I was thinking the man is the one [I would want to marry]. So told him but he scared he cannot afford, because want to take care of two of us. Because he actually cannot say the words [ask the girl to abort]. I think, you know, guys, so, I decide myself [to abort]”** (25yrs_Chinese_secondaryeducation_single_hairstylist)**

The spouse/partners also played a role in terms of supporting the women in obtaining abortion services. The support came in the form of moral and physical support such as accompanying the women to the clinic, seeking information on abortion, locating abortion services and paying or sharing the cost of abortion.

“Is really a tensión, sad right. I said in Islam it is a sin. I feel scared. I just kept my patience. Let him know [husband], shared with him. He said is not like want to do what, not that [we] purposely wanted this [pregnancy] right.it happened”** (22 yrs_Malay_secondary education_married_housewife)**

“My husband also searched in the Internet and he finds how many weeks is that? Is it a sin or not? He was looking at the Google and the Internet and all the stuff. He followed me to the clinic as well”** (29yrs_Indian_tertiary education_married_support specialist)**

“Umm…about the money, I have thought about it. I’ve discussed with my boyfriend and he helped me out with the money”*** (29yrs_Chinese_secondary education_single_admin clerk)***

Some women also received support from their mothers and good friends when the mothers took care of them, post-abortion, while they could confide their feelings and emotions with regard to the abortion with good friends.

“Er…my mum said because she old already. She also hope I can marry have baby. So she said if you can marry, no need to have abortion so I can be a grandmother. Yeah, it’s her wish but I said I don’t want she didn’t scold me. Also she took good care of me and cooked sesame oil chicken and ginger. Also good friend gave me support. Ya, I kept talking and telling them my feelings then she just say abortion is. She said abortion in the world, outside of the city and overseas, America, England there abortion is a very normal thing. Then she said no worry. She said never (become) too sad and upset your emotions. So, now I feel much better”*** (29yrs_Chinese_secondary education_single_admin clerk)***

### Needs of women in coping with abortion

The women highlighted two main abortion needs; abortion information and abortion services.

Many asserted that there is a need for abortion information to be disseminated to the public to increase public awareness and knowledge on this issue. Among the types of abortion information desired were the consequences or side effects, availability of the service, methods of abortion, abortion procedures and post abortion care. Most women had concerns about long term consequences of post-abortion but were not sure of the validity of the information and would like to receive detailed information about side effects.

“If let’s say we are pregnant, will there be effects on the child or what?”*** [*29yrs_Malay_secondary education_married_operator]**"

"“There are. like people said difficult to get pregnant again, is it true? I want to know more details about that”*** (*22yrs_Malay_secondary education_married_housewife)**

Need for information on abortion availability and accessibility were also highlighted by some women as some of them faced difficulties in locating or gaining access to the service. The women viewed that abortion information should be disseminated to the public to curb baby dumping as they reasoned that baby dumping is a greater sin and far worse compared to abortion.

“But in another way is better [abortion] then to throw the baby away right [baby dumping]. Abortion is already a sin that is being committed. If abort is a sin, throwing away baby is more sinful. Then the child has to suffer embarrassment. Is better like that [to abort]. If can, is okay to be disseminated [information on abortion availability]”** (29 yrs_Malay_secondary education_married_operator)**

“Because they all do all this [abortion], is wrong, but at least there are no baby dumping. There are no far worse and severe consequences”** (25 yrs_Malay_secondary education_married_clerk)**

Since the introduction of medical abortion was fairly recent, women expressed the desire to seek information about the side effects, safety, process, availability, effectiveness, ease of consumption of pills, cost, post abortion care and the advantages compared to other methods.

“The safety is the same [both surgical and medical abortion]. But don’t know. That’s why I feel because, when they do that [medical abortion] they didn’t use instrument. So may be is safer [compared to surgical abortion]. But we don’t know because there might be side effects inside [in the womb]. We don’t know about that. Because they never told us all these right? Like if we do this [abortion], what are the effects to our womb or if our uterus is ok or not ok”** (29yrs_Malay_secondary education_married_operator)**

Women felt that current abortion services are not easily found thus making it so difficult to obtain the service. Some viewed that abortion should be legalised by the Malaysian government in view of the occurrences of baby dumping and unsafe abortions which they had heard about.

“Em… I think government should give a way to provide legal abortion for Malaysian women. To make it less harmful for women who want to have abortion. But they can’t get the service legally, so they do it out there illegally. That can cause harm to their body. Can get the effect like won’t be able to get pregnant again, all the other effects”** (25yrs_Chinese_tertiary education_married_housewife)**

Pre- and post- abortion counseling were raised as an effective means to help women cope with abortion. Counseling could be used as a channel of delivery of detailed information about abortion and serve as psychological support to unearth their feelings and emotions so that women could relieve their anxiety about abortion. In addition, it was also mentioned that pre- abortion counseling could assist women in making informed decisions, i.e. whether or not to have an abortion.

“If me, there is a need [for counseling]. Like us, is it true when the doctor said there is nothing - like I always think, eh is it true? Is it really safe? Are there effects or not. Sometimes, after we wash [abort], why do we feel different? Feels like.always after we gave birth right. why is it that she looks different? Feel that she is bloated, why? Not from the effects of this [abortion] right? By right, he/she [doctor] should explain”** [22yrs_Malay_secondary education_married_housewife]**

“Like.what people said, counseling is okay because we talk to the person, chat with her to calm her down. Don’t give her tension. Counseling can help” [**22 yrs_Malay_secondary education_married_housewife]**

Future follow up following abortion were asked for by some women to safeguard the possibility of any long term side effects. One woman wished for more follow ups in case of long term effects of abortion.

“And then the follow up. Make sure our body is… is in good condition.”** (25yrs_Chinese_tertiary education_married_housewife]**

“The follow up, I’m quite dissatisfied with it because they only see ‘oh ok, now is clean nothing’. If can, let’s say we try after 3 months or 4 months we come back again [to the clinic]. We do the check up again. Like after [abortion] of course we would like to know the effects [of abortion] after 3 months or 4 months how. Scared if the effects will come right. With follow up, we can ask the doctor, the doctor will explain to us maybe because of imbalance or what”**(22yrs_Malay_secondaryeducation_married_housewife]**

## Discussion

This study found that most of the women seemed to favour medical abortion due to its convenience, naturalness and cheaper cost compared to surgical abortion. However there were concerns over the long term health effects of medical abortion and this, corroborates the study by Fielding (2002) [8]. Similarly, elsewhere, the acceptance and satisfaction towards medical abortion has also been reported in women [9-12]. Progression in medical technology have given women a safer, cheaper and simpler alternative namely, medical abortion. However, this method was noted to be uncommon in Malaysia as claimed by women in this study.

In Malaysian public hospitals, the main abortion method that is being carried out is still dilatation and curettage which is much less convenient, costlier and most crucially, riskier compared to manual vacuum aspiration (MVA) and medical abortion. The medical abortion regimen combining the use of mifepristone pills and misoprostol pills offers a complete abortion rate up to 95% in early pregnancies [13]. Although misoprostol could be given alone to induce a miscarriage, however, the effectiveness is still low in addition to a higher rate of side effects [13]. While mifepristone and misoprostol is registered in the WHO Model List of Essential Medicines [14] and is recommended to be used for early pregnancy termination [13], this is not the case in Malaysia. Mifepristone has yet to be registered while Misoprostol is only registered for use as a treatment for gastric ulcers. In Malaysia, the drug manufacturers need to make the initial move to register their drugs with the Drug Control Authority of Malaysia. This may not be forthcoming as for misoprostol, the manufacturer does not indicate it for O&G uses. Nevertheless, there should be efforts to overcome these difficulties.

The availability of both medical and surgical abortion should be disseminated and made available to women so that they can opt for a method that best suits them and at the same time, this choice would also give women control over their reproductive health matters [8].

Although the women in the study have undergone abortion, there were still reported fears and uncertainties towards abortion. The fears may have arisen from misperceptions about consequences of abortion and the lack of knowledge and awareness surrounding this issue. Abortion does carry complications such as infections and future sub-fertility and the women were aware of it. However, there were still some serious misperceptions such as: abortion could cause cancer and abortion would lead to birth of an abnormal child in future as reported in this study. This lack of knowledge could be due to the issue of abortion that is minimally discussed in the community. There has not been any provision of legitimate abortion information in the media or the healthcare services in Malaysia. If women were given information and were knowledgeable about the risks and consequences of abortion, perhaps the fears and worries could be avoided and also the misperceptions regarding side effects of abortion could be dispelled.

Access to abortion knowledge/information and services are the major barriers as evidenced from findings of this study. The inaccessibility could be multi-factorial from the clandestine nature of abortion, to cultural, spiritual and social barriers and also to lack of awareness to the abortion laws. In addition, the Medicines (Advertisement & Sale) Act 1956 (revised 1983) Act 290 states that “*No person shall take any part in the publication of any advertisement referring to any article, or articles of any description, in terms which are calculated to lead to the use of that article or articles of that description for procure the miscarriage of women*” [15]. Hence, abortion services are not widely or openly offered and are not easily accessible. These barriers caused women to” not know where and who to go for abortion” and they could only rely on information from friends or colleagues. In addition, discourse on abortion is rare and is viewed as a taboo. This makes it difficult for women to seek legitimate abortion information and its whereabouts. When women cannot and have difficulties in seeking abortion services, they resort to unsafe, self-induced methods [16,17] which is also admitted by women in this study. Such a scenario is alarming as self-induced abortion is harmful and could lead to severe complications [18] and even death [19].

Malaysian government hospitals do provide abortions but only under specific circumstances as permissible by the law as alluded above. However, this is not known by many women and including health providers themselves [20]. This could be another reason why women faced difficulties to obtain abortion services in Malaysia. It is pertinent that women and health providers must be made to know their rights to have, or to perform an abortion within the permissive provisions of the law so that it is clear to them that they are not committing a crime against the law. To women, they would then know their rights as a patient to have an abortion and would not have to resort to clandestine abortion services to which they could be subjected to harm. To health providers, knowing the abortion law would allow them to conduct their duties without apprehension so that they can provide the best quality care to patients within the law.

In this study, women expressed the difficulties they faced with health providers who disapproved of them having abortions by claiming that it was illegal. Efforts to increase awareness on the abortion law in Malaysia is warranted. In recent studies conducted in Malaysia on medical students and medical officers pertaining to their willingness to carry out medical or surgical abortion services, 41% of medical officers reported that they would not carry out abortion under any circumstances if it is against their personal religious beliefs (Mary Huang, Jegasothy & Lim, 2011. In Medical Officers’ Knowledge, Attitude And Willingness To Provide Abortion Related Service As Reproductive Rights Of Women, 2011; unpublished work), whereas it was less than 20% for medical students (Tey, Low, Yong et al., 2011. Survey on Knowledge and Perception of Medical Students on Abortion; unpublished work). This implies that many current and future health providers are and will not be willing to provide abortion services based on their personal and religious beliefs and this will cause serious repercussions when women are forced to continue an unwanted pregnancy.

Stigma attached to abortion is another challenge for women to have safe abortions and this is particularly for young, unmarried single women who are faced with an unwanted pregnancy [21]. This study found that the abortion stigma appeared at both individual and community level as the women revealed that they were embarrassed to ask for abortion information and also to go back to the same clinic for abortion because they felt shameful of letting people know they are doing a ‘wrong’ thing. In Malaysia, abortion is still considered a taboo and is culturally unacceptable for single or married women but more so for the former. These cultural beliefs hinder women from obtaining abortion information and services and drive women to have self-induced abortion or resort to unsafe clandestine abortion providers. Removal of stigma attached to abortion should be done so that women can get an unbiased, safe and quality medical treatment and care.

It is also found in this study that when women are determined to end their pregnancy, they are willing to pay whatever the cost. Such a situation would enable unscrupulous health providers to charge exorbitant prices for abortion services that is unaffordable by women from the lower socio-economic classes. For those who can’t afford to pay for the service, they will turn to cheaper alternatives such as traditional methods or seek illegitimate abortion services that are harmful which are usually available at a lower cost. Monitoring and regulation of abortion services is needed to avoid such events from occurring. However, for this to be done, clinics/healthcare centers which offer abortion services would need to come forward and make themselves known.

In terms of abortion decision making and support, the women in this study claimed to receive support from family members, friends and particularly from husband/partner. Men play a significant role in abortion as they have influence on the women’s abortion decision and emotional outcomes. Among the roles they play are as instigators, facilitators, collaborators, transporters, advisors, informers, supporters or punishment givers [22]. In terms of abortion decision making, although majority of the women in this study claimed to make the abortion decision by themselves but they had also sought their partner’s agreement or support and took considerations of their relationship with the partner before proceeding to have abortions. Kimport (2011) revealed that women felt the need to have control over their abortion decision and if the decision is not primarily theirs, they would experience emotional difficulties, however, it was further highlighted that support from partner, family and close friends were needed in order to validate their abortion decision making [23]. Other studies have shown similar findings where, when it comes to making abortion decisions, women had discussions with their partners, mothers and friends and were influenced by childhood experiences as well as their economic situation and future planning [24,25]. In the current study, husbands/male partners were also found to play a supportive role to the women in terms of providing moral support and help in accessing abortion services especially in terms of transportation and locating the service and financial assistance. Similar findings has also been found in other studies [26,27]. Support for women during pre and post abortion is crucial to avoid adverse physical and psychological effects [23,28] and attention is needed to be given to include men’s involvement in female reproductive and health matters.

Women’s needs with regards to abortion information and services are clearly highlighted in this study. Many of them are in favor for abortion information and services to be disseminated to the public. The lack of abortion information and services has prompted the call for increased availability and access to such services. Although there was favour for increasing and dissemination of abortion information and services to the public partly because the women themselves have had to use the service, another factor could be due to escalating incidences of baby dumping that is s a rising issue in the country at the time of data collection which indirectly influenced the women to feel the need for abortion services in the country. Perhaps, future studies can be conducted to determine the needs for abortion services in Malaysia in the general population and look into factors that lead to such a need, such as unmet contraception needs. The needs of women with regard to abortion uncovered in this study provided insights on the gaps that are existing in the current abortion services provided in the country and such needs should be addressed so that an accessible, comprehensive, safe abortion service can be provided.

### Strengths and limitations of study

This study has some strengths that should be noted. Firstly, based on the literature, it has been found that there is only limited research on abortion experiences and needs that have been carried out in the Malaysian context and none of these studies looked into women’s experiences with medical abortion and their needs with regard to abortion [29,30]. Hence, this study adds to the existing body of evidence for this topic in the country and to the internationally body of literature as a whole. Secondly, this study provided perspectives and empirical evidence into the topic of abortion which is often not talked about and discussed in Malaysia. Thirdly, the issues highlighted in this study will be of considerable importance for the understanding of the experiences and needs of women with regard to abortion in Malaysia and it is hoped that the findings can serve as a guide for developing policies and programmes to promote safe abortion which could potentially help to reduce dangerous clandestine abortion and ultimately reduce mortality of women.

We acknowledge that our study has limitations. Firstly, due to the qualitative nature of the study, the findings of this study cannot be generalized to the wider population. Secondly, the data gathered in this study required respondents to recall past experiences where recall bias was likely to occur. However, most of the participants had their last abortion less than one year from the period of data collection, thus the chances or recall bias were reduced. Thirdly, the respondents were recruited from only one urban clinic. Perhaps future studies could recruit women from various places (etc. clinics, healthcare centers, hospitals) from both rural and urban areas so that the understanding of the current abortion scenario in Malaysia could be captured from experiences of women from different settings.

## Conclusion

The existing abortion laws in Malaysia should enable the government to provide abortion services within the law. Unfortunately, the study findings show that this is generally not so, most probably due to the social stigma. There is an urgent need for the government to review its responsibility in providing accessible abortion services within the scope of the law and to look into the regulatory requirements for such services in Malaysia. This study also highlighted the need for educational efforts to make women aware of their reproductive rights and also to increase their reproductive knowledge pertaining to abortion. Besides the government, public education on abortion may also be improved by efforts from abortion providers, advocacy groups and related NGOs.

## Competing interests

The authors declare that they have no competing interests.

## Authors' contributions

WTT Conceptualization, acquisition of data, data analysis, manuscript write up. WYL Conceptualization, acquisition of data, data analysis, manuscript write up. YLW Conceptualization, acquisition of data, data analysis, manuscript write up. SPC Conceptualization, data analysis, manuscript write up. RJ Conceptualization, data analysis, manuscript write up. All authors read and approved the final manuscript.

## Pre-publication history

The pre-publication history for this paper can be accessed here:

http://www.biomedcentral.com/1471-2458/12/743/prepub
